# Supplementary material for: A Critical View of the Application of the APEX Software (Aqueous Photochemistry of Environmentally-Occurring Xenobiotics) to Predict Photoreaction Kinetics in Surface Freshwaters
Source: Molecules. 2019 Dec 18;25(1):9. doi: 10.3390/molecules25010009 (PMC7017383; doi:10.3390/molecules25010009)
Supplement: Supplementary file 1 [file molecules-25-00009-s001.zip › 3.2.4_gcc-4.4.0.zip!/3.2.4_gcc-4.4.0/3.2.4_gcc-4.4.0/doc/octave/html/interpreter/Getting-Started-with-Oct_002dFiles.html]

Getting Started with Oct-Files - Untitled


Next: Matrices and Arrays in Oct-Files,
Up: Oct-Files


---

#### A.1.1 Getting Started with Oct-Files

The basic command to build oct-files is `mkoctfile` and it can be
call from within octave or from the command line.

— Function File: **mkoctfile** [-options] file ...  
> The `mkoctfile` function compiles source code written in C,
> C++, or Fortran. Depending on the options used with `mkoctfile`, the
> compiled code can be called within Octave or can be used as a stand-alone
> application.
>
> `mkoctfile` can be called from the shell prompt or from the Octave
> prompt.
>
> `mkoctfile` accepts the following options, all of which are optional
> except for the file name of the code you wish to compile:
>
> -I DIR: Add the include directory DIR to compile commands. -D DEF: Add the definition DEF to the compiler call. -l LIB: Add the library LIB to the link command. -L DIR: Add the library directory DIR to the link command. -M --depend: Generate dependency files (.d) for C and C++ source files. -c: Compile but do not link. -g: Enable debugging options for compilers. -o FILE --output FILE: Output file name. Default extension is .oct (or .mex if –mex is specified) unless linking a stand-alone executable. -p VAR --print VAR: Print the configuration variable VAR. Recognized variables are: ``` ALL_CFLAGS FFTW_LIBS ALL_CXXFLAGS FLIBS ALL_FFLAGS FPICFLAG ALL_LDFLAGS INCFLAGS BLAS_LIBS LDFLAGS CC LD_CXX CFLAGS LD_STATIC_FLAG CPICFLAG LFLAGS CPPFLAGS LIBCRUFT CXX LIBOCTAVE CXXFLAGS LIBOCTINTERP CXXPICFLAG LIBREADLINE DEPEND_EXTRA_SED_PATTERN LIBS DEPEND_FLAGS OCTAVE_LIBS DL_LD RDYNAMIC_FLAG DL_LDFLAGS RLD_FLAG F2C SED F2CFLAGS XTRA_CFLAGS F77 XTRA_CXXFLAGS FFLAGS ``` --link-stand-alone: Link a stand-alone executable file. --mex: Assume we are creating a MEX file. Set the default output extension to ".mex". -s --strip: Strip the output file. -v --verbose: Echo commands as they are executed. file: The file to compile or link. Recognized file types are ``` .c C source .cc C++ source .C C++ source .cpp C++ source .f Fortran source .F Fortran source .o object file ```

Consider the short example

```
```
     #include <octave/oct.h>
     
     DEFUN_DLD (helloworld, args, nargout,
       "Hello World Help String")
     {
       int nargin = args.length ();
       octave_stdout << "Hello World has " << nargin 
             << " input arguments and "
             << nargout << " output arguments.\n";
       return octave_value_list ();
     }
```
```

This example although short introduces the basics of writing a C++
function that can be dynamically linked to Octave. The easiest way to
make available most of the definitions that might be necessary for an
oct-file in Octave is to use the `#include <octave/oct.h>`
header.

The macro that defines the entry point into the dynamically loaded
function is `DEFUN_DLD`. This macro takes four arguments, these being

1. The function name as it will be seen in Octave,- The list of arguments to the function of type `octave_value_list`,- The number of output arguments, which can and often is omitted if
       not used, and- The string that will be seen as the help text of the function.

The return type of functions defined with `DEFUN_DLD` is always
`octave_value_list`.

There are a couple of important considerations in the choice of function
name. Firstly, it must be a valid Octave function name and so must be a
sequence of letters, digits and underscores, not starting with a
digit. Secondly, as Octave uses the function name to define the filename
it attempts to find the function in, the function name in the `DEFUN_DLD`
macro must match the filename of the oct-file. Therefore, the above
function should be in a file helloworld.cc, and it should be
compiled to an oct-file using the command

```
     mkoctfile helloworld.cc
```

This will create a file called helloworld.oct, that is the compiled
version of the function. It should be noted that it is perfectly
acceptable to have more than one `DEFUN_DLD` function in a source
file. However, there must either be a symbolic link to the oct-file for
each of the functions defined in the source code with the `DEFUN_DLD`
macro or the autoload (Function Files) function should be used.

The rest of this function then shows how to find the number of input
arguments, how to print through the octave pager, and return from the
function. After compiling this function as above, an example of its use
is

```
     helloworld (1, 2, 3)
     -| Hello World has 3 input arguments and 0 output arguments.
```
